# Comparative mitogenomic and phylogenetic characterization on the complete mitogenomes of *Talaromyces* (*Penicillium*) *marneffei*

**DOI:** 10.1080/23802359.2016.1261610

**Published:** 2017-01-04

**Authors:** Emily W. T. Tam, Chi-Ching Tsang, Susanna K. P. Lau, Patrick C. Y. Woo

**Affiliations:** a Department of Microbiology, The University of Hong Kong, Hong Kong;; b State Key Laboratory of Emerging Infectious Diseases, The University of Hong Kong, Hong Kong;; c Research Centre of Infection and Immunology, The University of Hong Kong, Hong Kong;; d Carol Yu Centre of Infection, The University of Hong Kong, Hong Kong;; e Collaborative Innovation Center for Diagnosis and Treatment of Infectious Diseases, The University of Hong Kong, Hong Kong

**Keywords:** Mitogenome, *Talaromyces marneffei*, *Penicillium marneffei*, phylogeny, comparative genomics

## Abstract

We report the complete mitochondrial DNA (mtDNA) sequences of four *Talaromyces marneffei* strains and performed comparative genomic and phylogenetic analyses. The gene orders of the four mtDNAs are identical to the previously published mtDNA of strain PM1 (*nad4l, nad5, nad2, atp9*, *cob*, *nad1*, *nad4*, *atp8*, *atp6*, *nad6*, *cox3*, *rps*, *cox1*, *nad3*, *cox2*). Phylogenetic analysis showed that the four mtDNAs were clustered with that of PM1 with high bootstrap support. Compared to mtDNA of PM1, the only non-synonymous mutation was located in *nad2* (T505M) of strain PM26. Synonymous single nucleotide polymorphisms were observed at eight positions in the four mtDNAs.

*Talaromyces* (*Penicillium*) *marneffei* is the most important thermally dimorphic, systemic mycosis-causing fungus in Southeast Asia (Vanittanakom et al. 2006). Apart from being an AIDS-defining condition, penicilliosis has also been reported in other immunocompromised patients (Chan et al. 2016). Recently, we have described the emergence of penicilliosis in patients on anti-CD20 monoclonal antibodies or kinase inhibitors (Chan et al. 2015). In 2003, we reported the complete mitochondrial DNA (mtDNA) sequence of *T. marneffei* strain PM1 (Woo et al. 2003). Subsequently, we have reported its draft genome sequence (Woo et al. 2011), sexual cycle-related genes (Woo et al. 2006), and polyketide synthase gene clusters (Woo et al. 2010). In this article, we report the complete mtDNA sequences of four additional *T. marneffei* strains (PM18, PM19, PM20, and PM26) and performed comparative genomic and phylogenetic analyses. The four strains were isolated from penicilliosis patients in Hong Kong, and were deposited to the National Collection of Pathogenic Fungi (NCPF), UK (PM18 = NCPF 4320, PM19 = NCPF 4321, PM20 = NCPF 4322, and PM26 = NCPF 4323). Their mtDNAs were deposited in the International Nucleotide Sequence Databases with accession numbers KU761329–KU761332.

*Talaromyces marneffei* mtDNA was prepared from arthroconidia grown at 37 °C and purified using the Mitochondrial DNA Isolation Kit following manufacturer’s instructions (PromoKine, Heidelberg, Germany). The mtDNA was PCR-amplified using primers designed following the previously published PM1–mtDNA sequence (Woo et al. 2003), and the amplified products were sequenced with an ABI PRISM 3130*xl* Genetic Analyzer (Applied Biosystems, Waltham, MA). The sequences were assembled using CAP3 (Huang & Madan 1999). Annotation was performed according to Woo et al. (2003). The four mtDNAs were aligned with the mtDNA of PM1. Maximum-likelihood tree was reconstructed using MEGA 7.0.14 (Kumar et al. 2016).

The mtDNAs of the four *T. marneffei* strains are circular molecules, with lengths of 35,420–35,436 bp and G + C contents of 24.6–25.1%. The gene orders of the four genomes are identical to that of PM1 (*nad4l*, *nad5*, *nad2*, *atp9*, *cob*, *nad1*, *nad4*, *atp8*, *atp6*, *nad6*, *cox3*, *rps*, *cox1*, *nad3*, *cox2*). 63.6% of the four genomes are represented by structural genes (40.5% protein-coding exons, 5.9% tRNA genes, and 17.3% rRNA genes), 8.8% by intergenic spacer, and 32.4% by introns. They contain 15 genes encoding subunits of respiratory chain complexes (cytochrome oxidase subunits I–III [*cox1*–*cox3*], apocytochrome b [*cob*], reduced nicotinamide-adenine dinucleotide-ubiquinone oxidoreductase subunits [*nad1*, *nad2*, *nad3*, *nad4*, *nad4l*, *nad5*, and *nad6*]), three ATP synthase subunits (*atp6*, *atp8*, and *atp9*), and ribosomal protein of the small ribosomal subunit (*rps*). Phylogenetic analysis showed that the four mtDNAs were clustered with that of PM1 with high bootstrap support (Figure 1). Compared to PM1 mtDNA, the only non-synonymous mutation was located in *nad2* (T505M) of PM26. Synonymous single nucleotide polymorphisms were observed in PM18 at T1088C, T506C, C9268T, T11060C, and C11845T located in *nad5*, *cob*, *nad4*, *atp8*, and *atp6*, respectively; in PM19 at C11845T located in *atp6*; in PM20 at G1674A located in *nad2*; and in PM26 at G29088A located in *cox1*. The four mtDNAs also encode 28 tRNAs and the 23S and 16S rRNAs (*rnl* and *rns*).

**Figure 1.**
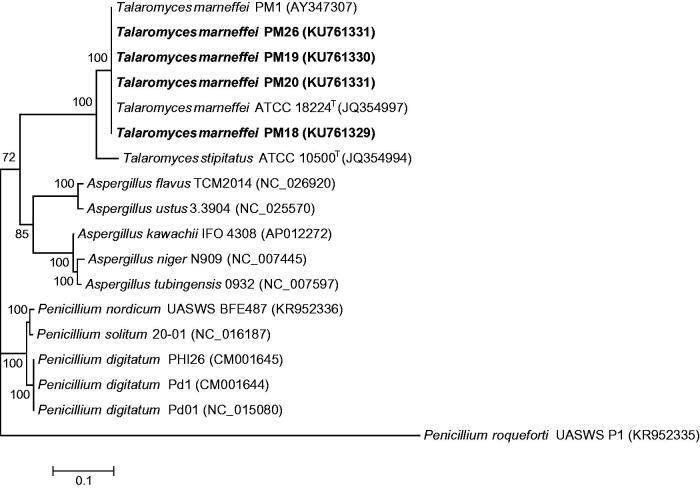
Phylogenetic tree showing the relationship of *Talaromyces marneffei* strains PM18, PM19, PM20, and PM26 to other *Aspergillus*, *Penicillium*, and *Talaromyces* species. The tree was inferred from the concatenated mitochondrial gene (*cox1*, *cox2*, *cox3*, *cob*, *nad1*, *nad2*, *nad3*, *nad4*, *nad4l*, *nad5*, *nad6*, *atp6*, *apt8*, and *atp9*) sequence data by the maximum-likelihood method with the substitution model GTR (general time reversible model) + G (gamma-distributed rate variation). The scale bar indicates the estimated number of substitutions per base. Numbers at nodes indicate levels of bootstrap support calculated from 1000 replicates. All names and accession numbers are given as cited in the International Nucleotide Sequence Databases.
